# Methodological issue in a dental neuromelioidosis outbreak investigation

**DOI:** 10.1016/j.lansea.2025.100658

**Published:** 2025-08-20

**Authors:** Bijayini Behera, Prasanta Raghab Mohapatra

**Affiliations:** a Department of Microbiology, All India Institute of Medical Sciences, Bhubaneswar, Odisha, India; b Department of Pulmonary Medicine & Critical Care, All India Institute of Medical Sciences, Bhubaneswar, Odisha, India

Thirugnanakumar and colleagues^1^ provided critical genomic evidence linking a neuromelioidosis cluster in Tamil Nadu to iatrogenic transmission via contaminated saline at a dental clinic.^1^ The identification of *B. pseudomallei* ST1553 with the *bimA*Bm_*Bm*_ allele in both clinical (brain tissue) and environmental (saline bottle) isolates unravelled a novel transmission route. The high fatality (80%) underscores severe neurovirulence concerns. While pivotal in revealing a novel transmission route, several aspects warrant further scientific discussion:

1. **Diagnostic gaps:** The low microbiological confirmation rate, 24% (5 of 21 samples), though consistent with global literature of low CSF culture positivity (<30%) in neuromelioidosis cases, is consistent with known challenges in culturing *B. pseudomallei* from CSF (<30%), highlighting inherent diagnostic difficulties.^2^
2. **Incomplete genomic data:** WGS of only two clinical and one environmental isolate from 5 available isolates (missing 3 clinical isolates) limits the robustness of the phylogenetic conclusion.
3. **Environmental sampling:** Positivity in only 1 of 28 samples (open saline bottle), combined with the inability to test dental water systems/instruments due to clinic closure, leaves potential contamination sources incompletely defined.
4. **Virulence interpretation:** Detection of BimA_Bm_ allele in both neurological as well as non-neurological isolates suggests its role in neurotropism may be more complex than suggested and requires further validation.
5. **Outcome confounding:** Significantly lower meropenem use in the dental-acquired cases (30% vs. 91%, p = 0.004) represents a plausible confounding factor contributing to the observed higher mortality, independent of transmission route
6. **Transmission mechanism:** While direct neural invasion remains hypothetical, hematogenous spread remains a viable alternative pathway that can’t be ruled out.
7. **Phylogenetic context:** Exclusion of 93% of global genomes (>2900 isolates) due to metadata gaps limits interpretation and constrains the broader interpretability of the outbreak strain's lineage.

While pivotal in exposing dental transmission risks, methodological constraints in confirmation, genomics, and environmental analysis necessitate cautious interpretation. Finally, this report calls for stringent IPC measures to prevent atypical and iatrogenic transmission routes of the deadly *B. pseudomallei.*

## Contributors

Dr Bijayini Behera- Conceptualization, writing (Original draft), writing (Review and Editing).

Dr Prasanta Raghab Mohapatra- writing (Original draft), writing (Review and Editing).

## Declaration of interests

None.
